# Relaxation versus exercise for improved quality of life in lymphoma survivors—a randomised controlled trial

**DOI:** 10.1007/s11764-020-00941-4

**Published:** 2020-09-28

**Authors:** Suchita Hathiramani, R. Pettengell, H. Moir, A. Younis

**Affiliations:** 1 Kingston University and St. George’s University of London, Cranmer Terrace, London, SW17 0RE UK; 2 St. George’s Healthcare NHS Trust, London, UK; 3 Kingston University London, Kingston Upon Thames, UK

**Keywords:** Lymphoma survivors, Exercise, Relaxation, Self-management, Quality of life

## Abstract

**Purpose:**

Lymphoma survivors experience persisting needs as a consequence of disease and treatment, which have an impact on quality of life (QoL). There is evidence supporting the use of relaxation and exercise to improve QoL, but there is no agreement on which is more beneficial. This study aims to compare a relaxation intervention versus an exercise intervention to determine which has a greater impact on QoL post-chemotherapy.

**Methods:**

Eligible participants (*n* = 46) were randomised to a relaxation or exercise intervention for 12 weeks. QoL was assessed at baseline, 6 weeks and post-intervention using the European Organisation for Research and Treatment of Cancer QoL Questionnaire Core 30 (EORTC QLQ-C30) questionnaire, which is a valid and reliable tool. The summary score and all EORTC domains were assessed.

**Results:**

There was a significant difference in QoL post-intervention between groups (*p* = 0.029) while adjusting for baseline QoL, with the exercise group demonstrating a larger improvement. Within-group QoL significantly improved pre- to post-intervention in both the relaxation (*p* = 0.036) and exercise (*p* = 0.004) groups.

**Conclusions:**

A self-management intervention of either exercise or relaxation can help significantly improve QoL in lymphoma survivors following chemotherapy. While exercise is preferred, a relaxation intervention would also have a beneficial impact on QoL.

**Implications for Cancer Survivors:**

Lymphoma survivors should be routinely screened and those with decreased QoL referred for an exercise programme, or relaxation for survivors who are unable to exercise or choose not to. A home-based programme can have a significant positive impact on QoL and is a feasible and effective method in the current climate.

**Trial registration number:**

Clinical Trials ID NCT02272751

## Introduction

It is well recognised that a large number of haematological cancer survivors continue to experience physical and psychological unmet needs following treatment, resulting in a decreased quality of life (QoL) [1–3]. It is also recognised that the immediate period following treatment is a particularly difficult time, when survivors may feel isolated and abandoned [4–6]. Several studies [7–10] have been carried out to assess the effectiveness of various rehabilitation interventions on QoL in cancer survivors. Some studies support the use of exercise such as aerobic and/or resistance training [7, 8], while others recommend the use of relaxation techniques including mindfulness-based stress reduction, progressive muscle relaxation and guided imagery [9, 10]. Both interventions appear to have a positive effect on QoL, but there is no consensus on which is more effective, and no standardised pathways following chemotherapy, despite the fact that cancer survivors report a need for further support during this transition phase [11]. In the majority of these studies, the relaxation or exercise interventions have been compared with a control group, and there has been a call for future studies to rule out potential placebo effects and compare with active control or other empirically supported interventions [12, 13], for instance relaxation to exercise.

Studies on cancer survivors have been carried out in various settings, including both group-based supervised programmes and self-management. Recent systematic reviews and meta-analyses of RCTs of exercise for cancer patients and survivors have reported significant beneficial effects for supervised exercise interventions on QoL and a lesser effect for unsupervised [14, 15]. This could be due to greater direction and motivation from a trainer and access to equipment. However, Swartz et al. [16] highlight that while there is evidence that exercise can improve the QoL of cancer survivors, few interventions have been translated into practice. This could be due to trial designs with high resources and clinical settings, and these authors suggest that home-based interventions may be a way of enabling the translation from research to practice [16]. Similarly, despite evidence of the efficacy of relaxation programmes for cancer survivors, the training and resources required to provide professionally administered psychosocial interventions can be a major barrier to their routine use [17]. Hence, although home-based programmes appear to have lower efficacy than supervised programmes, their greater accessibility, wider reach and potential long-term sustainability may make a self-managed approach also beneficial to survivors.

Furthermore, there is a drive towards self-management for cancer survivors, with the National Cancer Survivorship Initiative (NCSI) in the United Kingdom (UK) calling for a need to increase service user involvement and self-management [4]. Self-management can empower cancer patients and survivors, increase their confidence to manage problems associated with the disease and its treatment and enhance the quality of life [4, 18]. Survivors themselves have reported a preference for home-based self-management over group classes in a clinical setting [19–21]. In addition, due to the present COVID-19 pandemic, efforts are being made to reduce the exposure of cancer survivors to the virus [22]. Hence, effective, evidence-based self-management interventions are more pertinent now. The majority of survivorship studies have been carried out on the most prevalent cancers such as breast and prostate cancer [9, 10, 23], and there is a lack of studies on other cancer groups [1, 23] including haematological cancers such as lymphoma.

The Relaxation and Exercise In Lymphoma survivors (REIL) study was carried out to address some of these issues [24]. The REIL study aims to compare the effect of two home-based interventions on QoL in a group of lymphoma survivors within 6 weeks post-chemotherapy to determine which intervention—relaxation or exercise—results in a larger impact on QoL. The REIL study is the first randomised controlled trial (RCT) comparing home-based relaxation with a home-based exercise programme in lymphoma survivors following chemotherapy. Results will contribute to a better understanding of the efficacy of these interventions and build towards the implementation of evidence-based programmes to improve QoL in lymphoma survivors. This paper presents findings from the primary outcome measure, the European Organisation for Research and Treatment of Cancer Quality of Life Questionnaire Core 30 (EORTC QLQ-C30, version 3.0) [25, 26]. Secondary results will be reported elsewhere.

## Aim

To compare the effect of two home-based interventions (relaxation and exercise) on QoL in a sample of lymphoma survivors post-chemotherapy.

## Methods

### Study design

The REIL study is a randomised clinical intervention trial. Participants who consented were randomised to a relaxation or an exercise intervention. Outcome measures were assessed at baseline prior to intervention, at 6 weeks, and on completion of the 12-week intervention.

### Ethical approval

The study received ethical approval from Camden and Islington National Research Ethics Service (13/LO/1327), and from St. George’s Hospital Joint Research and Enterprise Office (JREO) (13.0108) where the research was carried out. The study is registered on a publicly accessible database, ClinicalTrials.gov, NCT02272751.

### Participants

Eligible participants included patients with histologically confirmed lymphoma in remission post-chemotherapy, chemotherapy treatment completed within the last 6 weeks, age 18 years or older, able to give informed consent, good performance status (assessed by the Eastern Cooperative Oncology Group [ECOG] status 0–2) [27] and medically able to undertake exercise. The following were exclusion criteria—patients with active disease, unstable angina or unexplained electrocardiogram, poor performance status (ECOG status 3 or more), pregnancy, difficulty breathing at rest, persistent cough, fever or illness, or any cognitive impairment limiting the ability to give informed consent or complete QoL questionnaires. Informed consent was obtained from all participants, and participants were informed that they could withdraw at any time. At baseline, patient demographics including gender, age, social history and medical history were recorded.

### Study settings

Participants were recruited from a single National Health Service (NHS) setting—the Haematology-Oncology Out-Patient (HOOP) Clinic at St George’s Hospital, London. Assessment for eligibility, recruitment, medical screening and obtaining informed consent was carried out by the patients’ medical consultant (RP). Assessment of outcome measures and delivery of interventions were carried out by the principal investigator (SH) following participants’ scheduled follow-up appointments.

### Interventions—relaxation and exercise

The relaxation intervention comprised an audio CD incorporating relaxation techniques including mindfulness meditation, deep breathing exercises, guided visualisation and progressive muscular relaxation. The exercise intervention comprised a programme of aerobic, upper and lower limb resistance exercise, core stability and stretches to be performed independently at home. Both interventions are described in detail in the REIL study protocol [24]. This protocol was adhered to throughout the study. Both interventions were similar in aspects including location (home), frequency and duration (50 min three times a week for 12 weeks). Participants in both groups also had equal contact with the researcher, approximately 3 h (1 h for each assessment and education session), and were able to call researcher for advice whenever needed. Both groups were provided with written information and resources including audio and visual aids to enable self-management. Participants in both groups were advised to continue to resume their normal activity as able, with their intervention as a supplement to normal habit [24].

### Outcome measures

The primary outcome measure is QoL, assessed by the EORTC QLQ-C30 summary score. The QLQ-C30 is a multidimensional health-related QoL questionnaire. This self-reported questionnaire is recognised as a valid and reliable tool and is the most widely used outcome measure used to assess QoL in cancer survivorship research [28]. Although the QLQ-C30 provides a wealth of information about QoL, a challenge is the multiple outcomes it generates. As a result, the EORTC group introduced and tested a single summary score of the QLQ-C30 questionnaire [29]. In addition to reducing the risk to type I errors, the use of the summary score can also ensure direct comparability between studies [29]. The EORTC group now recommends the use of the summary score and this was the primary outcome measure for the REIL study. The summary score and other EORTC domains were assessed at all three time points.

### Sample size

Sample size was calculated to determine clinically relevant effects on the primary outcome measure. A minimally important difference of 10 points in the EORTC QLQ-C30 score is generally accepted as clinically meaningful [30], and calculations were based on comparison of means between two groups. It was determined that a minimum 46 participants was required to detect a significant change in the EORTC summary score. This calculation assumed a two-sided significance level (*α*) of 0.05, power of 80% (*ß* = 0.20), a standard deviation from EORTC website reference values and a dropout rate of 34% as reported in similar studies [31]. Based on this, a minimum of 23 participants was required in each group.

### Randomisation

Participants were assigned to either the relaxation or exercise group using a computer-generated random allocation list. This was prepared independently by a biostatistician otherwise uninvolved in the study using randomisation software and saved on a secure database that could not be modified by the researchers. Each participant was assigned an anonymous ID number on enrolment, and each number allocated the intervention on the list.

All assessments were carried out by the principal investigator (SH); hence, it was not possible to blind the investigator to intervention. Participants also could not be blinded due to the nature of the intervention.

### Data analysis

Data was entered into the Microsoft Excel (2013) database by SH and analysed with support from the other researchers and a statistician independent of the study using IBM SPSS version 22 (SPSS, Inc., Chicago, IL) statistical software package. Summary scores plus scores from all other questionnaire domains were calculated as recommended by the EORTC scoring manual, missing data handled as recommended by scoring guidelines. Data was first assessed to check distribution using a combination of visual inspection, assessment of skewness and kurtosis and formal normality tests (Shapiro–Wilk). All data followed a normal distribution. Significance testing of baseline differences in a randomised sample is discouraged by CONSORT guidelines [32] and this was not performed; descriptive statistics are used to describe baseline characteristics of both groups. Analysis of covariance (ANCOVA) was used to compare differences in the QoL at 12 weeks using baseline QoL as a covariate. Post hoc paired-sample *t* tests for comparison pre-post intervention was tested within groups with the Bonferroni corrections.

## Results

### Participants

Participants were recruited from September 2014 to December 2016. Sixty-two potential participants were approached. Of these, 46 (29 female and 17 male) consented and were enrolled in the study, 23 randomised to each intervention. Participant demographics are summarised in Table 1.

**Table 1:** Baseline demographics

	Total sample, *n* = 46	Relaxation, *n* = 23	Exercise, *n* = 23
Age, years (mean + SD)	61 (+ 16.7)	60.4 (+ 19.4)	61.5 (+ 13.9)
Gender (*n*, %)			
Male	17 (37)	7 (30.4)	10 (43.5)
Female	29 (63)	16 (69.6)	13 (56.5)
Marital status (*n*, %)			
Married/partner	31 (67)	14 (61)	17 (74)
Single	10 (22)	4 (17)	6 (26)
Widowed	5(11)	5 (22)	0 (0)
Ethnicity (*n*, %)			
Caucasian	38 (83)	18 (78.3)	20 (87.1)
Asian	4 (9)	3 (13)	1 (4.3)
Afro-Caribbean	3 (6)	2 (8.7)	1 (4.3)
Other	1 (2)	0 (0)	1 (4.3)
Comorbidities (*n*, %)			
None	14 (30.4)	6 (26.1)	8 (34.8)
1	16 (34.8)	5 (21.7)	11 (47.8)
2	10 (21.7)	6 (26.1)	4 (17.4)
3 or more	6 (13.1)	6 (26.1)	0 (0)
ECOG status (*n*, %)			
0	7 (15)	4 (17.4)	3 (13)
1	18 (39)	7 (30.4)	11 (48)
2	21 (46)	12 (52.2)	9 (39)
Employment (*n*, %)			
Retired	21 (45.7)	11 (47.8)	10 (43.6)
Sick leave	9 (19.6)	4 (17.5)	5 (21.6)
Fulltime work	7 (15.3)	3 (13)	4 (17.5)
Homemaker	4 (8.6)	3 (13)	1 (4.3)
Part-time work	2 (4.3)	0 (0)	2 (8.7)
Unemployed	1 (2.2)	0 (0)	1 (4.3)
Other	2 (4.3)	2 (8.7)	0 (0)

**Fig. 1:**
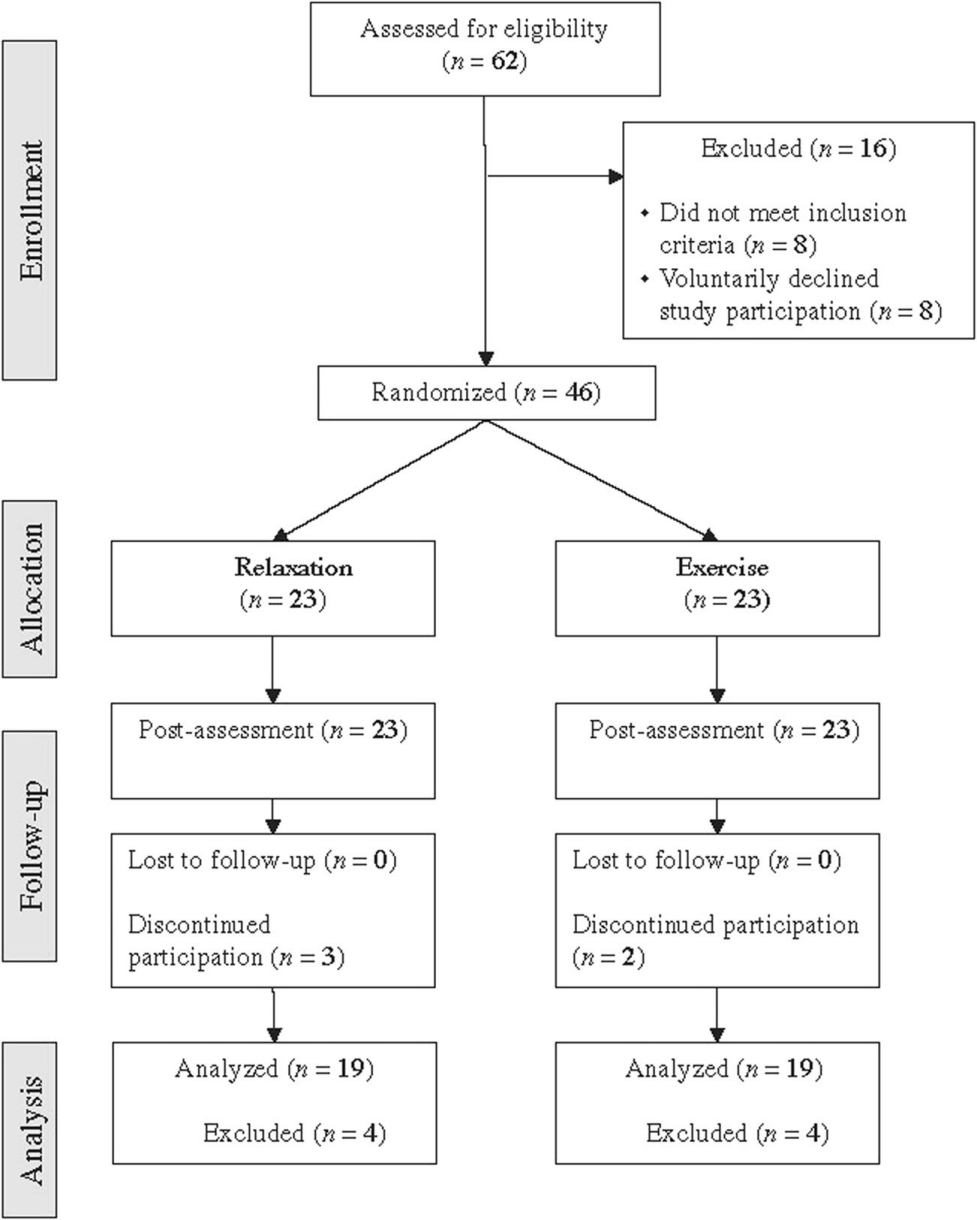
Participant flow

Five participants failed to complete the study (*n* = 3 relaxation, *n* = 2 exercise), reasons included no time (*n* = 3), not interested (*n* = 1) and disease progression (*n* = 1). This demonstrated a dropout rate of 13% for the relaxation group and 9% for the exercise group. Little’s MCAR test indicated that data were missing completely at random, and pairwise deletion for complete case analysis was applicable for this dataset. The CONSORT flow diagram of the study is presented in Fig. 1. No adverse events or injuries were reported in either group.

### Between-group effect—QLQ-C30 summary score

There was a significant difference in QoL between the two groups at 12 weeks (*F*(1, 35) = 5.208, *p* = 0.029; 95% CI 0.608–10.413) while adjusting for baseline. The effect size observed was large (partial eta squared = 0.130). The mean adjusted QoL summary score at 12 weeks was 91.07 (95% CI 86.69–93.58) for the exercise group and 83.68 (95% CI 81.18–88.07) for the relaxation group, indicating a beneficial effect of exercise vs. relaxation. Mean values of the summary score at 12 weeks and all EORTC domains are summarised in Table 2.

**Table 2:** EORTC QLQ-C30 domains—means (+ SD) of both groups and between-group comparison, adjusted for baseline. *Indicates significant difference between groups

	Mean 12-week relaxation	Mean 12-week exercise	*p=*
Summary score	83.69 + 11.34	91.07 + 7.03	0.029*
Physical function	77.54 + 18.02	89.21 + 11.03	0.038*
Role function	81.58 + 24.15	87.72 + 14.53	0.441
Emotional function	82.02 + 20.65	88.60 + 15.01	0.267
Cognitive function	78.07 + 16.71	86.84 + 17.19	0.367
Social function	87.72 + 17.43	92.10 + 17.01	0.444
Fatigue	35.67 + 22.40	17.54 + 18.63	0.009*
Nausea/vomiting	4.39 + 7.54	0.88 + 3.82	0.080
Pain	14.04 + 14.97	5.26 + 9.71	0.017*
Dyspnoea	19.30 + 23.08	10.56 + 15.92	0.093
Insomnia	17.54 + 23.22	5.26 + 12.49	0.155
Appetite loss	10.53 + 19.41	8.77 + 15.08	0.588
Constipation	10.53 + 19.41	8.77 + 15.08	0.588
Diarrhoea	7.02 + 17.84	3.51 + 10.51	0.342
Financial problems	8.77 + 24.45	7.41 + 18.28	0.911
Global QoL	74.12 + 15.93	80.70 + 15.48	0.374

### Other QLQ-C30 domains

There was a significant difference between the two groups for physical function (PF) (*F*(1, 35) = 4.642, *p* = 0.038; 95% CI 0.465–15.662), fatigue (*F*(1, 35) = 7.763, *p* = 0.009, 95% CI 4.339–27.640) and pain (F(1, 35) = 6.268, *p* = 0.017, 95% CI 1.610–15.418) subscales, adjusting for baseline values.

There were no significant differences between groups for other subscales.

### Improvement over time

**Fig. 2:**
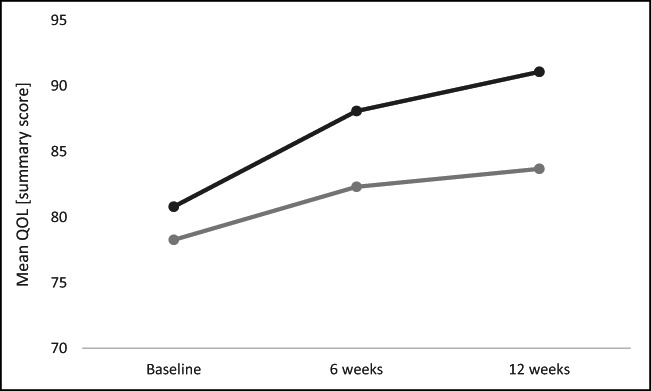
Mean QOL over time at baseline, 6 weeks and 12 weeks (dark grey line represents exercise intervention; light grey line represents relaxation intervention)

Within-group analyses demonstrated a significant difference in pre-post QoL summary score in both groups—mean difference pre-post in the relaxation group was 6.198 (± 11.92), *p* = 0.036, 95% CI 0.453–11.943; and in the exercise group, the mean difference was 8.839 (±11.563), *p* = 0.004, 95% CI 3.266–14.413. Within-group improvement in QoL from baseline to 12 weeks is demonstrated in Fig. 2.

## Discussion

The aim of this study was to compare the effect of two home-based interventions on QoL in a sample of lymphoma survivors within 6 weeks post-chemotherapy. Results indicate a significant difference between the groups following intervention, with the exercise group demonstrating a larger improvement in overall QoL than the relaxation group. A significant difference between groups in PF, fatigue and pain symptom scales was also observed, once again with the exercise group demonstrating a larger improvement in these domains.

Similar to this study, other studies on exercise in cancer survivors have similarly demonstrated a positive effect on QoL and other outcomes closely related to QoL such as PF, fatigue and pain [7, 14, 15, 33, 34]. Exercise has been shown to have a significant positive effect on physical factors including body weight, body mass index, muscle strength, aerobic fitness, fatigue and functional ability in cancer survivors, as well as psychological factors including distress, anxiety and depression [13, 32, 35, 36]. Hence, several national and international cancer organisations recommend exercise for cancer survivors [37–40].

Following these findings, it can be argued that a home-based exercise programme post-chemotherapy is more effective at improving QoL than a relaxation programme. It must be highlighted however that while the exercise group demonstrated a larger improvement, the relaxation group also demonstrated a significant difference in QoL following intervention. Other studies have also reported improvements in QoL following a relaxation programme [9, 41]. While the mechanism and physiology of relaxation are different from undertaking exercise, relaxation techniques have also shown to have a positive effect on physical and psychological symptoms in cancer survivors including pain, fatigue, quality of life, anxiety and depression [9, 41–43]. The use of complementary and alternative medicine (CAM) including relaxation techniques is growing in popularity, and it is reported that cancer survivors are more likely to use CAM than the general population [44]. Up to 89% of survivors report the use of CAM including mind-body approaches such as relaxation [45, 46], choosing to do so without being advised by a healthcare professional. Hence, a proportion of cancer and lymphoma survivors may have a preference for relaxation programmes. Hence, as the results of this study also indicate, the benefits of relaxation should not be overlooked, and both these interventions are beneficial to cancer survivors—relaxation could be considered an effective alternative intervention to offer to survivors who chose to or who are unable to exercise.

While studies have shown the effectiveness of mindfulness-based stress reduction (MBSR) on QoL, the traditional MBSR course requires a considerable time commitment and this could be a reason for non-adherence or even non-participation [47]. The authors have acknowledged there is scope to tailor the intervention so that it is less intensive, for instance, less time-consuming for the participant [47]. The relaxation intervention used in the REIL study meets criteria described for low-intensity interventions—administered in non-face-to-face setting, delivered by a non-mental health practitioner (delivered by a physiotherapist specialising in oncology), be more accessible (programme available in their NHS follow-up clinic) and be briefer for the practitioner compared to therapist delivered treatments [48]. Hence, this home-based relaxation programme is a feasible and realistic intervention for improving the QoL of lymphoma survivors unable to exercise.

It is recognised that cancer survivors suffering from comorbid conditions such as heart disease, hypertension, asthma/COPD, diabetes and osteoarthritis experience lower levels of QoL [49]. While exercise has a beneficial impact on a variety of these comorbid conditions, these survivors may require close monitoring and exercise under supervision. For this group, a self-managed home relaxation programme may help to improve their QoL, if they are unable to perform unsupervised exercise to do so, or experience any contraindications. Other groups of survivors may have a preference towards relaxation for a variety of other reasons including previous history of complementary and alternative medicine (CAM) use, or dislike of exercise. This group may also benefit from a self-managed relaxation programme if experiencing ongoing symptoms leading to decreased QoL.

Lymphoma survivors experience a heterogeneity of unmet needs [50]—while some complain of no needs following chemotherapy, others experience symptoms ranging from physical (e.g. fatigue, pain, nausea) and/or psychological (e.g. anxiety, depression, insomnia). This wide range of baseline needs complicate the provision of adequate support, and a flexible approach is required with opportunity to access different types of support at different times post-treatment [6], based on individual need and preference. It is widely recommended that interventions are tailored for individual patients and that patient choice should be a key feature in survivorship care [4]. As both the relaxation and exercise interventions improved QoL, offering a choice to patients who experience decreased QoL could be a way towards the flexible, tailored approach recommended for survivorship care. The main focus should be the assessment of survivors and identifying those in need of support to improve QoL.

As not all cancer survivors experience unmet needs and decreased QoL, some cancer survivors may not feel the need to undertake self-management practices as they feel they already have a good QoL [51]. This was seen in the REIL study, where some participants reported no unmet needs following chemotherapy and found no benefit from a home-based intervention [11]. The sample of lymphoma survivors in this study was already fairly active, as participants had to have an ECOG performance status of 0–2 [27] which ranged from fully active without restriction to mobile and capable of all self-care but unable to carry out work activities in order to be included in the study. Patients who were capable of limited self-care or confined to a bed or chair more than 50% of waking hours were excluded from the study; hence, the results could be affected by potential ceiling effects in the patient-reported outcome measure, where baseline QoL was a good score (higher score for summary score and function scales, and lower score for symptom scales) and thus less sensitive to change. More importantly, those with severe comorbidities resulting in a greater decrease in QoL and possibly in greater need of intervention (either relaxation or exercise) and support may have been excluded from this study if they did not meet performance status criteria or were unable to exercise independently. Hence, it is important to assess which survivors are most likely to benefit from an intervention programme, whether relaxation or exercise, and target those with the highest needs [52]. Screening for factors that negatively impact QoL such as comorbidity, moderate to severe symptom burden, psychological distress and fatigue should alert health professionals to a potential need for support and allow earlier intervention to improve QoL in those most affected [3, 53, 54].

As both the relaxation and exercise programmes demonstrated significant improvement in QoL post-intervention, another approach may be to integrate elements of both into one programme. Lee et al. [55] also compared two interventions in cancer survivors, one focusing on physical training (qigong) and one on mental (stress management), to a control group. These authors also found both groups demonstrated significant improvement in physical and psychological functions at 12 weeks compared with a control group, and also recommend that cancer survivors receive both physical and mental support as they can benefit from both. Multimodal interventions including both physical and psychological aspects have also been recommended by others [17, 19, 56]. Further study on the development of a multidimensional home-based intervention including aspects of both relaxation and exercise in a larger sample of lymphoma survivors is recommended in the future.

Lee et al. [55] report that although educational resources including DVDs and CDs were provided to encourage home practice, lack of persistence can result from lack of supervision and peer encouragement, and they recommend continuous and periodic involvement by professional coaches at a minimum of once a week. A qualitative study to explore gynaecological cancer survivors’ experience of a home-based exercise intervention also found that weekly telephone contact was favourable as it seemed to create an accountability to exercise, linked with a rapport with the contacting therapist [19]. In contrast, it has been stated that self-management in cancer survivorship should encompass understanding how and when to seek support, making lifestyle changes to promote health and wellbeing, and the onus should be on individuals to initiate contact with healthcare professionals [4]. This latter approach was adopted for the REIL study, using supported self-management during the transition phase. Here, while both the relaxation and exercise interventions were self-managed, participants were educated and advised to contact the researcher (healthcare professional with expertise in cancer rehabilitation) any time additional support or advice was required. In spite of evidence for the greater effectiveness of supervised programmes, cancer survivors report a preference for home-based and unsupervised exercise interventions for a variety of reasons for instance to avoid travel, time commitments, issues around cost, intimidation, and to avoid going back to hospital [20, 21, 57]. Interventions tailored to individual need and preference are essential for maximising recruitment and adherence and enhancing outcomes [21]. Home-based interventions can also include motivational support and guidance using distance-based approaches.

In the current climate of social distancing, distance-based interventions may represent a more practical and accepted method of support and rehabilitation for lymphoma survivors. While several studies on self-management and distance-based initiatives for cancer survivors including telephone counselling [58], mobile applications [59] and internet [60] have demonstrated significant positive effects including reduced physical side effects from treatment, reduced anxiety and depression, improved health behaviour and increased QoL, these interventions have faced barriers to implementation. Such barriers include not allocating resources to ‘new’ initiatives, unfamiliarity with the internet, reduced access to a smartphone and patient or healthcare professional attitudes (e.g. preferring face-to-face consultation or reservations about new technology) [22]. However distance-based strategies are now being promoted in most populations, in particular those at risk of serious complications from coronavirus, such as cancer survivors. Key organisations in the UK including the NHS England [61] and the Chartered Society of Physiotherapy (CSP) [62] now recommend distance-based assessment and rehabilitation as the primary strategy for cancer survivors where possible. Based on a high trial adherence, the absence of adverse events and positive satisfaction with the programmes [11], the home-based interventions in this study are considered feasible for lymphoma survivors in the transition phase. These interventions require comparatively low resources and personnel, incorporate the recommended elements of self-management, use of patient-reported outcome measures and tailored support when required, and have demonstrated a significant improvement in QoL; hence, such interventions would continue to be effective and practical even on the resumption of standard healthcare.

Strengths of the REIL study include testing of two theory-based interventions, use of patient-reported outcome measures and low dropout rates (17%). To our knowledge, this is the first RCT directly comparing the efficacy of two interventions on QoL; previous studies have used a comparison group as an active control group [63]. While it was not an aim of this study to directly evaluate the feasibility of the interventions, participants who completed their interventions in both groups reported at the end of the study questionnaire that they were able to fit this into their daily life [11]. Limitations include the small sample size. Difficulties were encountered with recruitment of eligible participants including relapse or disease progression, impact of lymphoma and its treatment, scheduling difficulties and technical problems. Similar problems have been reported in other studies in cancer survivors [64]. While this study did not include measures to assess and encourage participant adherence such as daily or weekly telephone calls or wearable activity trackers, the intervention programmes were designed to be carried out in ‘real-world’ settings and hence may be a more realistic representation of what lymphoma survivors are able to undertake. Also, no measures were taken to limit the practice of any other interventions in each group. Hence, participants in the relaxation group could have also taken up exercise on their own without being advised to, and vice-versa; this potential contamination could have biased results. However, once again, the addition of an intervention as a supplement to normal life without limiting other activities is a more realistic representation of real-world conditions. The potential ceiling effect of the EORTC QLQ-C30 questionnaire as discussed and the inability to stratify the sample due to small sample size for instance by physical activity are other study limitations. Another limitation is the predominance of female patients in the study sample. This gender imbalance has been reported in previous studies on interventions in cancer survivors— the authors have reported a clear majority of female participants from 73 to 87% [56]. This is in keeping with evidence that participation in population-based research is generally higher among women than among men [65]. Further research is needed to give insight into this gender difference, and there is a need for the development and testing of interventions with greater appeal to male cancer survivors [18, 65]. The principal investigator conducting both interventions also collected the data, which may have led to bias. Self-selection of participants may have resulted in a sample of lymphoma survivors who were motivated to undertake a self-management intervention, but this limitation is inherent in such studies.

## Conclusions

Lymphoma survivors may experience decreased QoL following chemotherapy and would benefit from assessment and referral for ongoing support and intervention as required. Based on this study’s findings, a home-based exercise programme results in a greater improvement in QoL than a relaxation programme. However, a relaxation programme would also have a similar effect, albeit to a lesser extent. Referral to either programme based on individual need and/or preference would aid in improvement QoL post-chemotherapy. Both interventions are an effective, feasible, less costly and more practical alternative to supervised group interventions in lymphoma survivors, particularly in the current climate of the COVID-19 pandemic and shielding of potentially vulnerable individuals.

## Implications for Cancer survivors

Self-management programmes with support as required are recommended for lymphoma survivors who demonstrate decreased QoL post-chemotherapy. A home-based exercise programme appears more effective than a relaxation programme in improving QoL and may be the favoured approach. However, for patients who are unable to exercise or choose not to, a home-based relaxation programme would also significantly improve QoL. Multidimensional programmes combining aspects of both may also be a way forward.
